# Implementing without guidelines, learning at the coalface: a case study of health promoters in an era of community health workers in South Africa

**DOI:** 10.1186/s12961-020-00561-5

**Published:** 2020-05-14

**Authors:** Teurai Rwafa-Ponela, John Eyles, Nicola Christofides, Jane Goudge

**Affiliations:** 1 Centre for Health Policy, School of Public Health, Faculty of Health Sciences, University of the Witwatersrand, Johannesburg, South Africa; 2 School of Geography and Earth Sciences, McMaster University, Hamilton, Ontario Canada; 3 School of Public Health, Faculty of Health Sciences, University of the Witwatersrand, Johannesburg, South Africa

**Keywords:** Health promotion, health systems, policy implementation, PHC reform, South Africa

## Abstract

**Background:**

Internationally, there has been renewed focus on primary healthcare (PHC). PHC revitalisation is one of the mechanisms to emphasise health promotion and prevention. However, it is not always clear who should lead health promotion activities. In some countries, health promotion practitioners provide health promotion; in others, community health workers (CHWs) are responsible. South Africa, like other countries, has embarked on reforms to strengthen PHC, including a nationwide CHW programme – resulting in an unclear role for pre-existing health promoters. This paper examined the tension between these two cadres in two South African provinces in an era of primary health reform.

**Methodology:**

We used a qualitative case study approach. Participants were recruited from the national, provincial, district and facility levels of the health system. Thirty-seven face-to-face in-depth interviews were conducted with 16 health promotion managers, 12 health promoters and 13 facility managers during a 3-month period (November 2017 to February 2018). Interviews were audio-recorded and transcribed verbatim. Both inductive and deductive thematic content analysis approaches were used, supported by MAXQDA software.

**Results:**

Two South African policy documents, one on PHC reform and the other on health promotion, were introduced and implemented without clear guidelines on how health promoter job descriptions should be altered in the context of CHWs. The introduction of CHWs triggered anxiety and uncertainty among some health promoters. However, despite considerable role overlap and the absence of formal re-orientation processes to re-align their roles, some health promoters have carved out a role for themselves, supporting CHWs (for example, providing up-to-date health information, jointly discussing how to assist with health problems in the community, providing advice and household-visit support).

**Conclusions:**

This paper adds to recent literature on the current wave of PHC reforms. It describes how health promoters are ‘working it out’ on the ground, when the policy or process do not provide adequate guidance or structure. Lessons learnt on how these two cadres could work together are important, especially given the shortage of human resources for health in low- and middle-income settings. This is a missed opportunity, researchers and policy-makers need to think more about how to feed experience/tacit knowledge up the system.

## Background

### Introduction

Health promotion (HP) and disease prevention have significant roles to play in reducing burden of disease by addressing key social, behavioural and other determinants of health [1]. In 2016, the Ninth Global Conference on HP recognised promoting health as significant to achieving the 2030 sustainable development goals [2]. Many have argued that HP needs to be afforded visibility and status at the highest level of government and the health system evidenced by governance, allocation of resources, and effective policies and programmes [3–5].

Over the last decade, there has been internationally renewed primary healthcare (PHC) focus [6]. PHC revitalisation is one of the mechanisms to emphasise preventive and promotive health in the public health agenda [7]. The 2008 World Health Organization (WHO) report stated that health systems have better outcomes when built on PHC approaches that have HP as a core component [6]. The early Ottawa Charter on HP noted the need for organisational change initiatives to provide valuable opportunities to ‘reorient health services’ towards prevention and promotion [8]. In this regard, it is essential to ensure that, within initiatives to reform PHC and efforts to strengthen health systems, HP is not just subsumed into PHC but also sufficiently acknowledged as part of the new PHC-focused reforms. Research has a potential role in influencing PHC reform policy formulation and implementation, through identifying possible factors for and against policy solutions such as these that seek to strengthen the health system [9, 10].

Despite the emphasis on HP integration into PHC, it is not always clear who should be leading its activities. Ideally, every health worker should do HP. However, in many low- and middle-income countries, there are shortages of nurses and doctors, especially in rural areas. If there is no specific cadre responsible for HP, it tends to get neglected [11]. Global health sector reforms have led to a re-emphasis of community health workers (CHWs). International literature has highlighted HP as one of the critical roles for CHWs [12–14]. CHWs are often the lowest level health workers, working primarily close to the communities they serve [12]. Different countries provide numerous examples for HP delivery, for example, in Canada, Australia and Guatemala, health promotion practitioners (HPPs) provide HP [15, 16], in others, such as Lesotho and Cambodia, CHWs are responsible for HP delivery [13, 14].

### South Africa

The outlining of HP in various policy and legislative frameworks evidences South Africa’s commitment. The 1997 White Paper on Transformation of Health Services highlighted the role of HP and health education [17, 18]. Within the bureaucratic structures of the Department of Health (DoH), there is a HP directorate at national level; each of the nine provinces have HP coordinators and some clinic facilities have health promoters [18, 19]. At the PHC level, the role of HPPs is to plan, implement and coordinate HP activities [18] such as health education, social mobilisation and outbreak response. In 2014, the national DoH finalised its first HP policy after being in draft for almost two and a half decades, together with a Strategic Plan (2015–2019) [20]. In addition, the White Paper on the upcoming National Health Insurance includes HP as one of its PHC service benefits, including the establishment of a multi-sectoral National Health Commission [21].

#### PHC revitalisation

South Africa, like many other countries, has embraced the concept of PHC revitalisation recommended by WHO in 2008 [6]. A series of strategies to strengthen PHC have been initiated. In 2011, the DoH adopted a three-main-streams approach to reform PHC, called ‘re-engineering of PHC’ (rPHC), comprised of district clinical specialist teams, ward-based outreach teams (WBOTs) and the integrated school health programme. The aim of rPHC is to shift PHC focus to a health-promoting community-based model [22, 23]. Deployment of community-based outreach teams is key in rPHC [22–24]. It propelled the ‘formal’ integration of CHWs into the district health system [22]. According to DoH policy documents, the team should ideally comprise of a professional nurse who is the team leader, six or more CHWs, an environmental practitioner, and a health promoter [24]. WBOTs are attached to a particular PHC clinic facility. Each team provides community, household and individual based health services within its catchment area [23, 24]. HP and disease prevention are part of the essential elements of CHW and WBOTs’ home visits and other community-based activities, such as screening, medication delivery and referrals [24]. The role of CHWs within WBOTs is considered central to achieving better health outcomes at the PHC level [25].

**Fig. 1:**
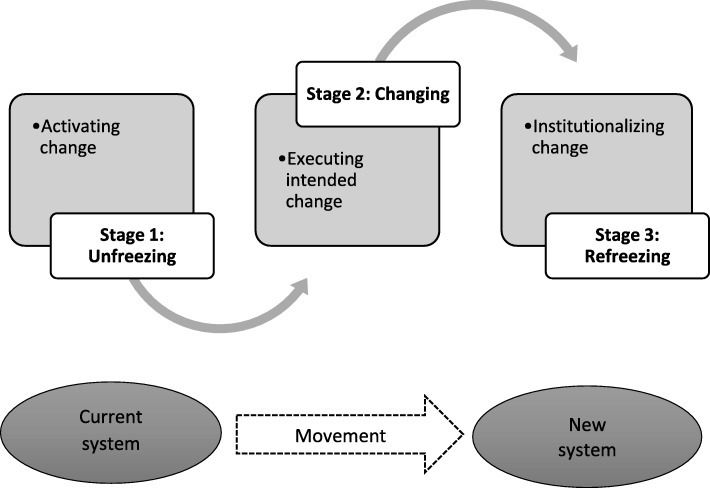
Lewin’s three-step change model, adapted from Cummings et al. [28]

Some areas in South Africa have health promoters and CHWs co-existing at the same clinic facilities, both doing HP activities. A gap in the literature exists on how HPPs are actively engaging with CHWs/WBOTs in practice, amid rPHC and the first HP policy documents. Therefore, herein, we examine the experiences of health promoters in the context of the introduction of CHWs and implementation of the rPHC WBOT strategy in two provinces in South Africa. Our focus was to understand how HPPs, as front-line policy implementers, are working with CHWs and WBOTs on the ground, in the absence of clear operational guidelines. We adopted Lewin’s organisational change theory [26, 27] as a lens to examine the effect of the introduction of the CHW programme among HPPs and the extent to which the two cadres are working together. Lewin postulated that organisational change occurs in ‘three steps’ – ‘unfreezing’, when existing structures are disrupted, preparing people for change, followed by change and, lastly, by new norms and practices and the creation of operating procedures [26, 27]. Although the three-step change theory has been criticised by other scholars for over simplicity [28, 29], it provides a useful framework to understand change in this study (Fig. 1).

#### Introduction of policy initiatives towards PHC reform

The introduction of rPHC and the HP policy were an impetus for institutional change in HP due to their unfreezing of the status quo of HP practice. The HP Policy and Strategy (2015–2019) [20], the first South African policy document to guide HP, was introduced at a time that the rPHC reform was being implemented. Table 1 shows text extracts from the national rPHC implementation guidelines [24] and the HP policy and strategic plan [20] describing  the role of HPPs in the context of CHWs/WBOTs.

**Table 1:** Health promoters’ roles and responsibilities in rPHC according to the two national documents of focus

Role of HPPs according to the rPHC implementation guidelines (2012) [24]	Role of HPPs in rPHC according to the HP Policy and Strategy (2014) [20]
Acknowledges the role of HPPs at community level and describes their role in PHC outreach teams, within the school health and in disease outbreak teams	“*Support CHWs with HP interventions*” and “*participate in school health teams*” *p. 25*
“*Ideally a health promoter is to support each PHC outreach team*” *p. 17.* This means that, all things being equal, one HPP would support one WBOT; however, this is not possible, as “*the availability of suitably qualified (B Degree in HP) persons may vary across provinces, districts, sub-districts and communities*” *p. 17*. Where they do exist, it is usually one per health facility	Promises to “*support health workers within the PHC WBOT to plan and implement community and social mobilisation efforts to meet the health needs of specific health systems and communities*” *p. 20*
Given lack of human resources for HP, where there is an HPP “*a health promoter could support 2–3 PHC outreach teams linked to a PHC clinic*” *p. 17*	“*Support the PHC outreach teams*” *p. 25,* under its implementation plans at district and sub-district levels
The rPHC implementation guideline states that “*the health promoter will provide overall support and technical assistance pertaining to HP to the PHC outreach teams*” and support to “*a. Develop and disseminate HP messages, b. Identify appropriate and relevant HP material for use and distribution, and c. use a range of HP tools*” *p. 17*	“*HP should be aligned with the rPHC programme in order to enhance, compliment and strengthen HP in communities, schools and health facilities*” *p. 26*
*“Assist and support CHWs by providing health information and updates on HP activities in accordance with the health calendar*” *p. 17*	The HP Strategy (2015–2019) promises to “*support PHC outreach teams to implement HP programmes*” *p. 30*

*CHW* community health worker, *HP* health promotion, *HPP* health promotion practitioner, *PHC* primary healthcare, *WBOT* Ward-Based Outreach Team

##### Policy content

Both national documents suggest a shift in HPP roles towards rPHC (Table 1), but provide limited descriptions of how health promoters are meant to provide support to CHWs and WBOTs. The rPHC guidelines mention HPPs supporting the WBOTs through developing and disseminating HP messages and identifying appropriate materials and tools; however, the HP policy has even less detail.

##### Policy implementation

Even though national policy documents for rPHC and HP are in place, practices on the ground remain unclear. Despite the rPHC reform highlighting the need for HP and preventive services in South Africa [24], there has been no formal process undertaken to re-orient the role of HPPs or re-aligning their job descriptions to include engagement in rPHC. The rPHC guidelines assume HPPs have higher levels of education than what currently exists for many. HPPs made no mention of developing and disseminating messages with or to the CHWs. Instead, they reported not having information, education and communication materials themselves, so would be unable to support WBOTs in this regard.

## Methods

Content analysis approach was used to underpin the study. The study was conducted from a relativist perspective, using the social constructivism theory to understand a social reality. The paper presents findings of a qualitative study that examined the context of changing the approach to HP in PHC in South Africa from the case study of the implementation of CHWs. Interpretivists believe that reality can only be understood through people’s subjective lens and interpretation of lived experiences [10]. We assumed that there are multiple perspectives of health promoters’ roles at the PHC level [30]. Thus, we recruited participants using a whole system view (national to facility level). The research feeds on health promoters and managers’ (HP and facility) views and beliefs and how they provide meaning to HP and rPHC [10, 30].

### Study design

We used a qualitative case study research design in two provinces in South Africa. This design was selected because it enabled an in-depth understanding of the roles of health promoters at the PHC level, particularly in the context of PHC revitalisation. A case study approach allows for an empirical inquiry that investigates a contemporary phenomenon within its context, especially when the boundaries between the phenomenon and context are not clearly evident [31]. In this study, the case of interest was the impact of the rPHC WBOT strategy implementation on health promoters; we describe their roles and activities (the ‘what’) are and the ‘how’ they are working with CHWs [32]. The data were collected during a 3-month period (November 2017 to February 2018). Forty-one participants were interviewed and two main national policy documents on rPHC and HP were reviewed. Data for this paper were collected as part of a mixed-methods research aimed at answering the research question ‘how is HP institutionalised within the South African health system?’ The research design of the larger project embedded quantitative data into a fundamentally qualitative approach [QUAL (quan)].

### Researcher characteristics and reflexivity

At the time of the study, the first author (TR) and principal investigator (PI) was a doctoral research fellow. TR conducted all the interviews and initial document reviews. Study participants did not know TR prior to conducting this research. TR has an HP qualification and previous experience in HP, although her professional training and experiences were mainly from another country, thus enabling her to assume the role of an outsider. Her background in HP made her more openly received, particularly by HPP participants. However, she found that some of the HP challenges highlighted by participants during the research resonated with her own experiences. In this regard, she consciously found herself making efforts to assume her role as the researcher versus being the practitioner and trying to keep an objective mind throughout the research process.

### Study sites

The study took place in two districts of two selected provinces that offered different contexts, experiences and views regarding HP and health promoters to ensure maximum variability on findings [33]. Twenty-one study sites were purposefully selected to represent various levels of the health system: national (*n* = 1), provinces (*n* = 2), districts (*n* = 2), sub-districts (*n* = 4) and facilities (*n* = 12). Names of provinces, districts, sub-districts and facilities have been anonymised. The selection of the two provinces was based on the availability of DoH health promoters practising at local PHC levels; not all provinces have designated HPPs at lower levels (district to clinics). Of the two provinces, Province A represented a mainly rural context, while Province B represented a largely urban setting. The two districts included were selected based on having the highest number of HPPs compared to other districts in each respective province. In the same regard, sub-districts and clinics were selected depending on the availability of an HPP during data collection.

### Sample, sampling and sample size

#### Description of participants

A purposive sample of HP and facility managers and health promoters were recruited. Specific personnel were requested from each site for inclusion. Multi-stage purposive sampling was used to recruit the participants into the study. This was done to represent all levels of the health system for maximum variability. At each level, the HPP participant identified would assist in identifying and recruiting other participants from the next level (district, sub-district and PHC clinics, respectively). TR used direct face-to-face invitations to recruit participants. Thirty-seven interviews (34 individual and 3 groups) were conducted, with 41 interviewees. Three of the interviews were group interviews conducted with 2–3 participants. Group interviews were conducted when more than one potential participant was present at a site and all were willing to participate in the study. We could not exclude one over the other. The first author made the decision to interview these participants in one interview, which enriched the interviews conducted. CHWs and WBOT leaders were excluded from this study. We felt that facility managers were able to provide a non-HP perspective to the study data. Facility managers directly supervise clinic-based health promoters, with the aid of HP coordinators at sub-district level. Table 2 shows the participant recruitment procedure for the study, from the national to the local level.

**Table 2:** Category and number of participants in each province (*n* = 41)

**Location/level of interviewees**	**Number of interviewees**	
National level HP management	6 (with 1 group of 3 interviewees)	
	**Province A**	**Province B**
Provincial level HP management	2	1
District level HP management	1	1
Sub-district level HP management	3	2
PHC clinics/facility level		
Health promoters	5	7 (with 1 group of 2 interviewees)
Facility managers	6	7 (with 1 group of 2 interviewees)
**Total interviews for each site**	**17**	**18 (with 2 groups of 2 s interviews)**

*HP* health promotion, *PHC* primary healthcare

#### Units for document review

In addition to the participants described above, two national policy documents were included in this study and specifically reviewed, namely the rPHC implementation guidelines (2012) [23] and the HP Policy and Strategy (2015–2019) [1].

The researchers used their knowledge of the availability of these national documents and guidelines. We regarded these two main policy documents as the most relevant for this study compared to any other, and purposefully selected them for inclusion in the research. The two documents are publicly available. We retrieved copies of them online, through searching for their titles via the Google web search engine and downloaded them for analysis.

### Data collection

#### Policy document review

National policy documents on rPHC and HP were reviewed to understand the role of HPPs in the context of CHWs and the primary health reform. The two main documents were selected based on their significance to HP practice and rPHC in South Africa. The first author read the full policy documents, and extracted content that spoke to the intent of policy direction of HP on rPHC or vice versa for inclusion in the study [34] (Table 1 shows the policy extracts that were relevant for this paper). In addition, the HP and rPHC text segments from the HP policy and strategy documents were compared to rPHC implementation guidelines to seek for alignment and/or identify any gaps. This process was done to have a baseline against which to assess the health promoters’ practices in rPHC and HP.

#### Participant interviews

This paper draws on face-to-face in-depth interviews using a semi-structured interview guide [35]. The purpose of the interviews was to inform the researchers about how participants understood the role of HPPs, and the way in which these understandings shaped health promoter practice within the WBOTs strategy of rPHC [36]. In-depth interviews were chosen as the main method to collect data for this study, as they allow collection of comprehensive and complex data about a participants’ feelings, thoughts, perceptions and experiences [37]. During the study, most interviews were conducted from the participants’ workplaces. The study participants themselves selected the space to be interviewed, which they considered private. We utilised DoH offices, for HP managers (apart from one that was done at the PI’s office, as the participant had come for another meeting in the area), and clinics staff rooms and/or consultation rooms for PHC facility-based staff (except for one which was conducted in the PI’s car due to lack of space and privacy at the clinic). Interviews were audio-recorded using both a mobile device and a recorder. The PI took down field notes during the interviews in a notebook. Interviews lasted for an average of 1 hour, ranging from 30 minutes to 2 hours. Before an interview commenced, participants were provided with an information leaflet and were requested to complete consent and sociodemographic forms. We did not receive any refusals to participate. One clinic was excluded from the study as the health promoter was not feeling well; although both the health promoter and the facility manager were willing to participate in the study, another clinic facility had to be chosen instead. All interviews were audio-recorded and transcribed verbatim. Audio files were de-identified. Anonymity and confidentiality of the study participants was ensured. Data collection ended when the targeted sampling frame and data saturation were reached. Data saturation was considered as the point at which we had sufficient data to answer the research questions, coupled with no new data expected to emerge from further collection of data [38]; data collection was stopped at this point.

##### Interview guide

The first author developed the interview guide in English, under the supervision of the third author (NC). The research team used their personal experiences in qualitative research and their knowledge of HP in the South African context together with the literature to guide development of the instrument. The interview guide was pre-tested on a single HP expert at the principal investigator’s institution (this interview was retained and used in other parts of the broader research, not reported in this paper). No changes were made after the pre-test. However, the instrument was adjusted depending on participants’ job location, level and role, which resulted in six variations of the evolving tool (national to facility). Topics covered by the instrument broadly included: (1) pre-questions, about the position and the participant’s role; (2) introductory questions, about how HP is implemented within the DoH; (3) HP policy questions, about the vision and strategy of HP within the DoH; (4) questions about HP successes, challenges, facilitators and barriers to implementation; (5) rPHC questions, about the role of HP and health promoters in PHC revitalisation; and (6) closing questions, retrieving participant perceptions about the future of HP in South Africa.

### Data processing and analysis

The authors conducted both inductive and deductive thematic content analysis, including descriptive statistics of participants’ characteristics. An independent transcription company transcribed audio-files from the interviews verbatim. TR verified for consistency against their original recording. A random sample of transcripts was shared with all research team members to familiarise themselves with the data. The codes developed were discussed and revised by all the members of the research team during consultative meetings, where necessary changes were made. This was done to elicit similar meanings of codes among each research member. MS Word files of transcripts were imported into MAXQDA 2018 software, which supported coding together with their original audio-files for comparison. TR performed the primary data coding. NC was involved in the review of codes and sub-themes. During the inductive content analysis, each interview transcript was read and text segments were coded based on emerging themes. Codes were then categorised into broad themes [39]. In this paper, two major themes and their categories are described. Firstly, we presented the general role and purpose of HP at PHC level. Under this theme, four main categories emerged, namely (1) purpose of HP practice; (2) settings for HP; (3) HP roles; and (4) types of information used to prioritise HP activities. Of these, 16 codes emerged. It is important to note that some participant phases’ cut across a number of codes and sub-themes. Secondly, we described HP and CHWs in the context of rPHC and the WBOT strategy.

Throughout the data collection phase, we had explicitly asked participants to discuss the role of HPPs in rPHC. Therefore, in the deductive process, we specifically coded transcript text segments [40], which spoke or referred to the second key theme – CHWs, WBOTs or rPHC. This particular theme was also used to extract text segments during the document analysis. Six key categories emerged under this theme, namely (1) role overlap; (2) CHW programme; (3) anxiety among HPPs; (4) working in parallel; (5) training of CHWs by HPPs; and (6) working together. However, other sub-categories, such as the school health programme and district clinical specialist teams’ strategy, which spoke to rPHC, were not included for analysis in this paper. We had an iterative process of going back to data to confirm emerging themes for both the inductive and deductive approaches. Lewin’s theory was identified once the categories started to emerge from the data. We then returned to the data to confirm the usefulness of the framework in explaining the data. Therefore, the three-step change domains were used to deductively analyse and present our findings, particularly under the second key theme of rPHC to unpack the ‘what’ and ‘how’ of HP and CHWs at the coalface. However, in-between the two key categories, an intersecting category emerged – introduction of policy. Thus, 23 sub-themes emerged in this paper. The quotations provided were selected on the basis that they either exemplified a common viewpoint among participants or provided unique information [36]. Table 3 shows an example of the audit trail for data coding.

**Table 3:** Examples of an audit trail used to move from participant phases to themes

Participant phases	Categorisation		
	Codes	Categories	Themes
1. My role is to assist the community in preventing diseases … (BP011HPP)	Disease prevention	Role of HP	Health promoters at PHC level
2. We network with other institutions, NGOs and stakeholders … (BP001SD)	Stakeholder engagement	Purpose of HP practice	
3. Health promotion goes around in schools and crèches … (AP015FM)	Schools	Settings for HP	
4. If there is a malaria outbreak, clinic stats show us that and we intervene …. (AP006SD)	Clinic stats	Prioritisation of HP activities	
5. Aspects of what CHWs do is HP, they give health education …. (ND002)	CHWs vs. HPPs	Working as part of one team	CHWs and the WBOT rPHC strategy
6. If there is something new that we’ve learnt, my job is to train them [CHWs] (AP014HPP)	Train or capacitate		
7. When they [WBOTs] encounter some challenges … we go and see what the problem is … (BP007HPP)	Collaboration		

*CHW* community health worker, *HP* health promotion, *HPP* health promotion practitioner, *PHC* primary healthcare, *rPHC* re-engineering of primary healthcare, *WBOT* Ward-Based Outreach Team

## Results

In our findings, we first describe our study participants and then follow Lewin’s three stages of change model to unpack the organisational change process that occurred after the introduction of the new policy initiatives.

### Study participants

Participants were HP managers operating at different levels in the health system (39.0%, *n* = 16), health promoters (29.3%, *n* = 12) and facility managers (31.7%, *n* = 13). The sociodemographic characteristics of participants are displayed on Table 4. About half of the participants were from the PHC level (61%, *n* = 25/41).

**Table 4:** Sociodemographic characteristics of participants (*n = 41)*

Variable	Classification	Frequency (***n***)	Proportion (%)
**Sex**	Female	32	78.1
	Male	9	22.0
**Age in years**	< 25	1	2.4
	25–34	2	4.9
	35–44	11	26.8
	45–54	17	41.5
	55+	10	24.4
**Race**	Black	39	95.1
	White	1	2.4
	Indian	1	2.4
**Highest level of education**	Some secondary	1	2.4
	High school	4	9.8
	Certificate	4	9.8
	Diploma	15	36.6
	Bachelor’s degree	9	22.0
	Post-graduate	8	19.5
**Job title**	Health promoter	5	12.2
	HP practitioner	10	24.4
	HP coordinator	4	9.8
	HP liaison officer	2	4.9
	Facility manager	13	31.7
	Other	7	17.1
**Job location**	National	6	14.7
	Province	2	4.9
	District	3	7.3
	Sub-district	9	22.0
	PHC/facility	20	48.8
	Other	1	2.9
**Years worked at current job location**	≤4	4	7.8
	5–6	3	7.3
	6+	34	82.9
**Years worked as current job title**	≤4	5	12.2
	5–6	4	9.8
	6+	32	78.1
**Previous job title**	None	5	12.2
	CHW/Care giver	2	4.9
	HIV counsellor	1	2.4
	HP manager	6	14.6
	Others	27	65.9

*CHW* community health worker, *HP* health promotion, *PHC* primary healthcare

### Stage 1: Unfreezing the status quo

#### HP practice and general roles and responsibilities of health promoters at PHC level

Most participants identified health education as a core function of health promoters, using words like health talks, giving information, teaching and/or awareness giving to describe it. Health promoters play an important role in the community (where they are expected to spend up to 80% of their time) conducting door-to-door and school visits. HPPs have a role in disease prevention; statements such as “*prevention is better than cure*” were often used, mainly focusing on the healthy lifestyle programme. Health promoters also formulate support groups, and coordinate and facilitate health awareness events. Participants often referred to conducting community outreach as ‘social mobilisation’. All HPPs expressed the need to establish relationships with stakeholders in one’s catchment area, including participating in clinic committees. During this phase, we witness the introduction of the policy initiatives. Box 1 summarisers the general roles and responsibilities of health promoters at PHC level.

### Stage 2: Change

#### Extent of role overlap between health promoters and CHWs

Despite HP being commonly recognised as a pillar of PHC by participants, none readily offered information about the HPP role in WBOTs. Only after being specifically prompted was such information provided.

Some HPPs described how they do similar roles:

“ *… we do similar things with WBOTs. They visit households. When they visit a house, they ask if anyone has been coughing for more than two weeks. As a health promoter, you must do that.*” (AP007HPP health promoter)

Door-to-door visits were the most common denominator between CHWs and health promoters. A provincial HP manager affirmed this view by stating:

“*… a health promoter is on the ground; one of their strategies for health awareness is door-to-door? So now, HP and CHWs are both doing door-to-door.*” (AP016P HP manager)

“*… when you look at the CHW programme what would you call it? It’s also HP. So now, we’ve got duplicated HP activities within the country.*” (AP016P HP manager)

Another HP manager emphasised the importance of HP at PHC level, and expressed that part of what CHWs do is HP:

“*HP is a foundation. You can’t have PHC without HP. Although we may not be formally recognised as such. But, I mean if you look at the entire CHW programme, it’s the basis of PHC. Aspects of CHWs’ work is HP: they are doing health education, working in communities, providing support, being mediums in which messages are disseminated. That to me is a pure form of HP, at that level and PHC can’t function without that.*” (ND003 HP manager)

One of the district HP managers regarded the rPHC concept as not new because HP was already doing some of its aspects:

“… *re-engineering for me, it's not a new concept, except the definition of the Minister.*” (BP015D HP manager)

Referring to how HP has always concentrated on working in communities and identifying clients in need. Therefore, change led to some role duplication.

Some participants identified the differences between the two cadres. Conversely, the change may have led to different work emphases. HPPs were reported to visit schools and other community settings, whereas the CHWs are limited to working within households, except during community campaigns. One HP manager explained:

“*Health promoters are more of your community facilitator type, coordinators, identifying problems, deal with issues, refer …*” (BP016P HP manager)

HP ‘asks why health problems exist’ while CHWs link patients to health services:

“*CHWs focus more on ensuring that people are linked to services. They go out and track people that have defaulted. Instead of asking why, are people defaulting? With HP, you ask why so many people are defaulting? How can we intervene? CHWs go out and check how many households are there. But, if there is overcrowding, what is it that they do? Can they facilitate linkage with other services? They cannot. But that’s what health promoters do, because they work with other sectors and link with other stakeholders.*” (BP015D HP manager)

One HP manager explained:

“*I see CHWs performing more clinical work, as they are the outreach programme of the clinic, following up on non-attending patients and those who have just been discharged from hospital.*” (BP001SD HP manager)

This viewpoint was common among some HPPs:

“*HP is only different with CHWs because they diagnose; they take vital signs* [blood pressure and sugar checks] *at home.*” (BP007HPP health promoter)

However, CHWs do not diagnose patients, and rather simply record measurements on behalf of the clinic.

#### CHWs receive more attention and resources

Upstream HP staff were not involved in rPHC planning or prioritisation. Most HP managers and HPPs felt that the CHW programme had received more attention and capacity strengthening than its “*HP relative*”:

“*If you know you have a cadre named CHWs, and you know their functions are related to HP. When you’re making changes or skilling, you should make sure that these* [HP] *programmes are attended to equally, but that never happened.*” (BD015P HP manager)

Some facility managers affirmed this lack of political support for HP:

“*… you’ll notice that even our management at higher level* [DoH]*; they actually don’t recognise HP as an important programme. It has no political will, neither is it allocated any budget.*” (BP006FMAB facility manager)

This view of lack of recognition for HP by the DoH was common among all study participants. The focus on the CHW programme highlighted how change causes discomfort among implementers.

Mid-level HP managers affirmed that the introduction of CHWs was received with high political support:

“*… sometimes you will find CHWs being considered as a programme that is actually above the HP programme.*” (AP001SD HP manager)

Others emphasised how CHWs were given a particular training, whilst HPPs had not received any targeted DoH training. The national DoH has not yet set competency levels for HP in South Africa. One HP manager explained:

“*… but, when you look at the resources and the training that is given to these people* [CHWs]. *Now you ask yourself, why can’t we do the same with HP and even put them at a certain level.*” (BP015D HP manager)

The feeling of HP being left out was common among HP managers in this study:

“*… and now health promoters don’t even have some of the skills that CHWs have, like blood pressure* [measurement].” (AP016PHPmanager)

The desire expressed by mid-level HP managers was for health promoters to perform at a level higher than CHWs. Some HP managers described some confusion brought about by the training of CHWs, who receive certificates describing their qualification as one in HP:

“*CHWs are actually being registered as HP officers, what bearing does this now have on the health promoter?*” (ND004ABC HP manager)

Such feelings point to disruption caused by change.

#### Anxiety among some HPPs

The introduction of WBOTs led to anxiety and trepidation among some HPPs. Mention of CHWs was met with mixed feelings among HP managers and facility-based HPPs alike:

“*… you know it brought some level of fear and uncertainty on the part of health promoters.*” (BP015D HP manager)

This included fear of losing their jobs. The same HP manager commented:

“*… remember when it was introduced, it was so prominent. It was pushed so hard, and pitched at a level that would undermine what already existed* [HP]*. So it was like is HP being phased out? It brought a level of uncertainty.*” (BP015D HP manager)

This particular HP manager, however, described how they held meetings within their district to address this uncertainty. They explained how rPHC fitted with broader PHC goals, and clarified the concept of working in a *‘ward’*:

“*… so, at least because it’s been years now and nobody has lost their job, HPPs are comfortable.*” (BP015D HP manager)

A few mid-level HP managers did not support the introduction of CHWs, nor did they understand why CHWs were introduced. As a result, they believed that there was no need to introduce these “*new cadres* [CHWs]”, given that HPPs were already in existence, arguing that someone higher-up the DoH ranks should have advocated for more HPPs instead. Perceptions regarding the possibility of merging CHWs and HPPs under one directorate were common among some HP managers:

“*I think they were supposed to be auxiliary health promoters instead. Like in social work, we have a social worker* [and] *auxiliary social workers.*” (ND004ABC HP manager)

Since CHWs were now doing household visits, one facility manager suggested limiting health promoters’ work to facilities:

“*… now that we have got CHWs, why can’t we have them focus on communities and HPPs focus on the facility?*” (BP012FM facility manager)

### Stage 3: refreezing into ‘new’ practice

#### Working in parallel

HP managers raised the issue of HPPs and CHWs running parallel HP activities, one district HP manager commented,

“*Now when you look at CHW duties, it’s to go identify, screen and refer. But, what happens to the issues that they identify within the community? That is where HP comes in. Now you look at their reporting and how those issues are brought up. It’s parallel. So, it means, a health promoter will just find a way to work and they end up addressing issues that they think are important. But, when you look at it, as we create these other structures* [CHWs]*, we create parallel working situations whereas we want to act on issues in the same area.*” (BP015D HP manager)

In addition, the same district HP manager pointed out that the HP role of CHWs is compromised given that the facility manager and the WBOT leader are clinicians, suggesting that the refreezing may be out of kilter:

“*… remember when they brought in the coordinator* [WBOT] *at the facility, it’s a nurse. This person is very much clinical. At the end of the day, they do not move on the same understanding* [with HPP]*. Therefore, that will make HP in the facility and catchment area run in parallel with CHWs, because these two also do not link. It’s the facility manager, the health promoter here; and a WBOT coordinator there* [illustration]*. These two, facility manager and the WBOT leader are clinical. They would understand each other. Automatically, that would frustrate implementation of HP and how the health promoter operates.*” (BP015D HP manager)

#### HPPs training CHWs

Notwithstanding the lack of clear guidelines and duplication, some health promoters embraced their role in rPHC. Facility managers regarded HPPs as critical in the initial stages of rPHC rollout. They described how health promoters had informed people in the community about WBOTs and the CHW programme, and encouraged them to be warmly received. A HPP explained:

“*I work with WBOTs because I know these people well. They are my patients. I started working here earlier, and I know them better. But, I was not providing them with medication* [like CHWs do]*. I was arranging for them to come to the clinic every month to collect the tablets. When WBOTs came, I took them around, to show them patients who are in need.*” (AP007HPP health promoter)

Another facility manager confirmed this view:

“*Without HPPs telling clients and the community about all the services coming with PHC re-engineering, it won’t succeed. They are the ones that are supposed to go in front of us, before we can come…*” (BP003FM facility manager)

One common response among study participants was the in-service training of CHWs:

“*… 80% of what CHWs do may be health education and a bit of HP, but mostly health education. It should be a health promoter that guides them on how to do it.*” (ND003 HP manager)

CHWs were regarded as not having enough health information, relying on HPPs for it:

“*CHWS don’t have enough information; they rely on health promoters to train them. Some things are changing like the PMTCT; you will find that something that was true five years-ago is no longer practical now. So whenever we get new information we also give it to CHWs…*” (AP002D HP manager)

In this way, HPPs were enacting a facilitation and support role during the refreezing phase of the CHW programme after the uncertainty and confusion that was expressed about the introduction of the new programme.

#### HPPs working as part of WBOTs

At some health facilities, HPPs were reported to work as members of WBOTs, although this is informal:

“*I am part of the WBOT. I don’t do the household registrations* [like CHWs]*. But, I do patient follow-ups with them and visit households with CHWs when they have a problem…*” (BP002HPP health promoter)

One sub-district HP manager confirmed this information:

“*HPPs are part of the WBOTs and remember they are community health workers* [literal meaning]*.*” (AP010SD HP manager)

However, the involvement within WBOTs varied per health promoter. HP managers described the frequency of HPP visits within WBOTs as determined at facility level:

“*HPPs are not always with WBOTs, depending on the needs of services at the time. Yes, there are instances whereby the health promoters and the WBOTs go together and conduct door-to-door services, but there are instances whereby they are not able to…*” (AP010SD HP manager)

Health promoters reported task sharing when they visit homes as part of WBOTs, their role being to give *“deeper”* health information. HPPs also regarded themselves as having a supervisory role over CHWs:

“… *it’s like we’re working as supervisors of the WBOT. Even if I’m not presenting myself as a supervisor, I am working together with them. There is a lot of work, and it is easier now than before because CHWs are present on the ground with the people and stay in that community.*” (AP002HPP health promoter)

Many HPPs frequently cited “*stepping in*” or lending a hand when CHWs face problems in the community. They mentioned that CHWs are expected to report health-related issues they face during their work in the community to HPPs. Some health promoters were reported to sit together with the WBOT leader and go through CHWs’ reports to find where there are needs for HP interventions:

“*She is doing households together with the WBOT. CHWs and HPP give information at households and bring patients to the facility. That makes a great improvement in case management of conditions. Because nurses will not go to households to trace* [patients]*. But, health promoters together with CHWs are the ones that visit households and trace patients that are not getting services, market the services and refer them to the facility.*” (AP005FM facility manager)

Most HP staff expressed how they have grown to appreciate and embrace the presence of CHWs at PHC level, some stating that the introduction of CHWs has *“been a blessing in disguise”* because a lot of work which was on the HPPs’ shoulders has been lessened, especially household visits.

## Discussion

Herein, we provide insight on how the HPPs’ role has been challenged in two provinces in South Africa by the introduction of CHWs. The rPHC and the first national HP policy were introduced and implemented without clear guidelines on how health promoters were to provide support to CHWs and WBOTs. Despite considerable role overlap and the absence of formal re-orientation processes to re-align HPP roles with rPHC goals, some informal integration between HP and CHW/WBOT activities has occurred on the ground. However, there is also some role confusion, with the two cadres, and their associated programmes, working in parallel. In Table 5, we use Lewin’s change framework to summarise facilitators and barriers to jointly working together [26, 27]. Although change processes are iterative and do not occur in clear stages, policies and other changes initiated from the top-down can lead to unfreezing of everyday practice. Change brings about some sort of discomfort and refreezing is subject to individuals and context specific factors.

**Table 5:** Change in this study using Lewin’s three-stage model

**Stage**	Organisational action that occurred	Factors for or against working together	
		Facilitators	Barriers
**Unfreeze**	Policy-makers introduce rPHC and the HP policy and launch the new reform	• Health promoters know communities well	• Limited re-alignment of HPPs for new roles: ◦ job descriptions ◦ re-training
**Change**	HPPs and CHWs/WBOTs experiment on how to work together on the ground	• HPPs as forerunners of the CHW programme	• Role overlap • More attention and resources towards CHWs • Anxiety and trepidation among HPPs
**Refreeze**	Some HPPs institutionalise new change into their practice culture	• HPPs train CHWs • Working at part of one WBOT	• Two cadres, and their management structures, working in parallel

*CHW* community health worker, *HP* health promotion, *HPP* health promotion practitioner, *rPHC* re-engineering of primary healthcare, *WBOT* Ward-Based Outreach Team

Staff from the HP programme were not involved in the development of the rPHC implementation guidelines. We found that the rPHC change vision was not well communicated between policy authors, national DoH, and front-line implementers - HPPs at district level; with better communication, HPPs might not have perceived CHWs as a ‘threat’. Our findings are similar to a New Zealand study, which found that health promoters felt vulnerable after the introduction of a strategy to shift HP into primary health organisations [41]. No previous local or regional research could be found that had examined HP or its human resources relative to a PHC reform. Since implementation of the rPHC policy, there has not been any new guidance on the roles and responsibilities of HPPs, nor has the national HP directorate developed new job descriptions for facility-based HP staff. Our findings mirror those of Hill [42], who examined how written policy often fails to provide implementers with sufficient detail to perform policy change, meaning that HPPs and other cadres of the health system must implement new policies with limited guidance. It is not surprising, therefore, that the refreezing was not uniform but varies according to characteristics of provinces, districts and health providers (e.g. facility managers and HPPs). Research shows that policy and guidelines are not always adequately implemented [43–45]. This study adds an important piece to the puzzle by highlighting the challenges faced by front line workers in implementing guidelines that provide insufficient ‘guidance’.

In contrast, there has been tacit learning at facility level with no mechanism for that learning to be transmitted ‘up the system’. Findings from this coalface experience of HPPs could be used to inform the development of new job descriptions and operational guidelines for HP practice and strengthened rPHC implementation. Examples from South Africa exist where learning from implementing change enabled policy design for sexually transmitted infection management and antiretroviral roll-out – where policy implementers at the bottom influenced policy elites at the top, through continual communication and networking among policy-makers, practitioners and researchers during the implementation process, influencing national uptake [46, 47]. Study findings highlighted in this research represent a missed opportunity for learning from the bottom-up. We learn that some sufficiently skilled and confident health promoters have carved out a role for themselves, supporting the CHW programme, particularly where supervision from the local facility is lacking. Our results provide further support for the hypothesis that implementation of policy change is at the discretion of front-line implementers [48, 49]. However, global experiences show that combining both top-down and bottom-up initiatives is crucial for effective implementation of reforms [50–52]. Research findings presented herein can also help fill these gaps by shaping policy through linearly feeding this experience/tacit knowledge up the system [53].

Our findings suggest that CHW HP activities fall by the wayside. Given that, facility managers and WBOT leaders who oversee them have primarily clinically oriented duties. They are more likely to neglect the non-curative aspects of CHWs in rPHC. In an earlier South African study, CHWs did not necessarily see themselves as ‘health promoters’, because they did not view health education as an important service to deliver [54]. A study in Lesotho revealed that the ability of CHWs to perform HP activities has been heavily affected by their role to increase health service access [13]. Many HP managers would have preferred CHWs to be merged under the same HP directorate. Yet, Lehman and Gilson, in their paper on power practices in implementation [55], describe how the provincial HP directorate in the Western Cape province of South Africa lost the battle to be in charge of the CHW programme to the provincial HIV directorate, which had access to external funding. Power dynamics influence change implementation [56]. Those directorates with power over resources, for example, the HIV directorate, are more likely to be heard and respected.

Studies on rPHC in South Africa have mainly concentrated on CHWs and WBOTs. Research conducted in Gauteng and the Eastern Cape provinces found none of WBOTs under study included an HPP [57, 58], although the Eastern Cape study found 4 out of 9 WBOTs were supported by an HPP [57]. This different interpretation of a vacuum left by national guidelines shows that provincial imperatives might dominate implementation.

Low investment in HP or its workforce is a global phenomenon [59–61]. A south Australian study, found unintended consequences of top-down policies to strengthen HP in PHC led to fewer opportunities for it, with HPPs reporting experiences of contradictory policy and practice environments, including funding and policy directions prioritising individual behaviour change [34]. Some countries have initiated formal educational, professional and/or competency standards for HP [4, 62, 63]. In Guatemala, HPPs are community based, often with low levels of education [16], while in others, like Australia, Canada, Israel, Botswana and Zimbabwe, HPPs have relevant HP qualifications [15, 62, 64–66], and in Mexico it is a combination of both categories [67]. In Mexico, given a lower than ideal supply of HPPs to deliver HP services at the PHC level [11], the two groups of health promoters have been separated out – professional and non-professional community based [67]; this model may have relevance for South Africa. In Box 2 below, we provide some recommendations for South Africa using lessons learnt from our study.

There are several limitations to this study. Firstly, the research was conducted in two out of nine provinces. However, with 41 in-depth interviews, our findings and conclusions are transferrable to other parts of South Africa. Secondly, no CHWs/WBOT leaders were interviewed, although facility managers represent a neutral non-HP voice in the study. Lastly, we did not conduct focus group discussions and observations of HPP’s work at facility or community level to validate information given and to enable added triangulation of findings.

## Conclusion

Our study presents a case study of what happens in the absence of proper guidelines to implement organisational change introduced through top-down initiatives, and how those at the bottom made sense of the proposed change. Some of the HPPs managed to find a role for themselves introducing CHWs, on-going community engagement, training and supervising the CHWs. With formal role descriptions, a greater number of health promoters could take on these primary health reform roles.

Box 1 Summary of HP practice and general roles and responsibilities of health promoters at PHC level as reported by study participants **Settings for health promotion:**

• Working in communities, health promoters are not clinic/facility bound

 ○ visit households

  ▪ trace defaulters

  ▪ follow-up visits

 ○ schools, early development centres/crèches

 ○ churches

 ○ workplaces

 ○ public spaces, e.g. bus ranks, beer halls, marketplaces

**Purpose of health promotion practice:**

• Disease prevention, they work with ‘preventive measures’ compared to curative

• Promote healthy behaviours through the healthy lifestyles programme

 ○ nutrition

 ○ physical activity

 ○ substance abuse, e.g. alcohol and tobacco

 ○ salt reduction

 ○ safe sex (e.g. condom demonstrations)

• Other programmes/topics covered include:

 ○ HIV/AIDS adherence to medication

 ○ Tuberculosis (early diagnosis and adherence to medication)

 ○ Prevention of mother to child transmission

 ○ Early antenatal care booking

 ○ Hand washing

 ○ Malaria prevention

 ○ Promoting screening for non-communicable diseases, i.e. diabetes and high blood pressure

 ○ Listeriosis (was most recent topic during time of study)

**Health promotion roles:**

• Provide health education on health topics, such early pregnancy booking, HIV, tuberculosis, prevention of mother to child transmission, nutrition, hygiene, substance abuse, etc. (a core function)

 ○ giving information, teaching, preaching and/or health awareness

• Community outreach often labelled as ‘social mobilisation’

 ○ door-to-door campaigns

 ○ community-wide campaigns

• Campaigns/events planning

• Stakeholder engagement

 ○ buy-in

 ○ mobilise resources

 ○ refer patients/clients for non-health services

• Formulating and facilitating support groups

 ○ age-group specific

 ○ gender specific

 ○ disease specific

• Being part of clinic committees

• Other roles and responsibilities include:

 ○ behaviour change

 ○ outbreak response

 ○ clinic-community relationship building

 ○ understand community health needs

 ○ patient/client advocacy

 ○ strengthen community participation and community empowerment

 ○ distribute condoms and information, education and communication material if available

 ○ manage clinic help-desk

 ○ participating in community meetings

• Working in communities, health promoters are not clinic/facility bound

 ○ visit households

  ▪ trace defaulters

  ▪ follow-up visits

 ○ schools, early development centres/crèches

 ○ churches

 ○ workplaces

 ○ public spaces, e.g. bus ranks, beer halls, marketplaces

**Sources of information for prioritising health promotion activities:**

• Community profiling

• Clinic/health facility statistics

• Health calendar days

• Outbreaks

Box 2 Recommendations from the study Policy-makers and HP managers (at the top), can learn from innovation within facilities (at the bottom) and develop formalised operational guidelines and direction for HPP routine practices, particularly within the primary health reform.

Role clarification of HPPs using a combination of approaches:

 • Re-framing the role of HPPs to that of a more senior level, in which their role is to support, supervise and train CHWs, in line with qualifications set in the rPHC implementation guidelines

 •Developing job descriptions, including formal integration of health promoters into WBOTs and conducting patient follow-up and household visits with CHWs

 •Re-training HPPs across the health system (national to PHC) to be able to provide formal support and oversight to CHWs and WBOTs

 •Defining competency levels for the HP workforce in South Africa to be able to train and recruit appropriate cadres to fit into the goal of the primary health reform

## Data Availability

Datasets used and/or analysed during the current study are available from the corresponding author on reasonable request.

## Abbreviations

CHW: community health worker
DoH: Department of Health
HP: health promotion
HPP: health promotion practitioner
PHC: primary healthcare
rPHC: re-engineering of primary healthcare
WBOT: Ward-Based Outreach Team
WHO: World Health Organization
